# Is there potential for the future provision of triage services in community pharmacy?

**DOI:** 10.1186/s40545-016-0080-8

**Published:** 2016-09-29

**Authors:** Louise E. Curley, Janice Moody, Rukshar Gobarani, Trudi Aspden, Maree Jensen, Maureen McDonald, John Shaw, Janie Sheridan

**Affiliations:** School of Pharmacy, Faculty of Medical and Health Sciences, University of Auckland, Private Bag 92019, Auckland, 1142 New Zealand

**Keywords:** Pharmacist, Community pharmacy services, Triage, Advice, Referral, Primary health care, Patient outcome assessment

## Abstract

**Background:**

Worldwide the demands on emergency and primary health care services are increasing. General practitioners and accident and emergency departments are often used unnecessarily for the treatment of minor ailments. Community pharmacy is often the first port of call for patients in the provision of advice on minor ailments, advising the patient on treatment or referring the patient to an appropriate health professional when necessary. The potential for community pharmacists to act as providers of triage services has started to be recognised, and community pharmacy triage services (CPTS) are emerging in a number of countries. This review aimed to explore whether key components of triage services can be identified in the literature surrounding community pharmacy, to explore the evidence for the feasibility of implementing CPTS and to evaluate the evidence for the appropriateness of such services.

**Methods:**

Systematic searches were conducted in MEDLINE, EMBASE and International Pharmaceutical Abstracts (IPA) databases from 1980 to March 2016.

**Results:**

Key elements of community pharmacy triage were identified in 37 studies, which were included in the review. When a guideline or protocol was used, accuracy in identifying the presenting condition was high, with concordance rates ranging from 70 % to 97.6 % between the pharmacist and a medical expert. However, when guidelines and protocols were not used, often questioning was deemed insufficient. Where other health professionals had reviewed decisions made by pharmacists and their staff, e.g. around advice and referral, the decisions were considered to be appropriate in the majority of cases. Authors of the included studies provided recommendations for improving these services, including use of guidelines/protocols, education and staff training, documentation, improving communication between health professional groups and consideration of privacy and confidentiality.

**Conclusion:**

Whilst few studies had specifically trialled triage services, results from this review indicate that a CPTS is feasible and appropriate, and has the potential to reduce the burden on other healthcare services. Questions still remain on issues such as ensuring the consistency of the service, whether all pharmacies could provide this service and who will fund the service.

## Background

The demands on primary health care services worldwide are growing [1], largely due to an ageing population which has subsequently led to increased strain on the primary health care workforce [2–5]. In order to overcome such challenges, primary health care systems have evolved to encompass new services and, in many countries, extended roles for community pharmacists [6, 7].

Triage has traditionally been described as the sorting and allocation of treatment to casualties, particularly in battlefield and disaster situations [8]. In this model, casualties are sorted based on a system of priority, designed to maximise the number of survivors [8]. The definition has been extended to refer to “The assessment of patients on arrival to decide how urgent their illness or injury is and how soon treatment is required” [9]. An example of the latter description includes the role of nurses in emergency rooms [8]. More recently, the term triage has been used increasingly to describe non-emergency situations in healthcare: one such example is Healthline in New Zealand, where members of the public can speak to a registered nurse who provides advice and directs patients to the most appropriate service [10].

Community pharmacy is recognised for its role as a common first port of call for patients in the provision of advice on minor ailments [11], and referral to an appropriate health professional when necessary [11]. Community pharmacies are available in most localities, often open at times when general practitioner (GP) services are not available, and no appointment is necessary to consult with a pharmacist [4, 5]. This raises the question of whether there is an opportunity to translate the concept of triage to a formalised service provided by community pharmacists.

It could be argued that elements of triage services in community pharmacy already exist. Worldwide, a number of medicines have been reclassified from prescription-only medicines to be available over-the-counter, as medicines available only from pharmacies [12]. Examples include chloramphenicol for the treatment of bacterial conjunctivitis [13] and trimethoprim for uncomplicated urinary tract infections [14] in New Zealand. This reclassification enables appropriately trained pharmacists to determine when to treat and when to refer the patient to their GP or other health professional, and thus includes an element of triage, although the skills and processes used to undertake this task are not currently referred to in this way.

Developing effective triage services in community pharmacy has the potential to reduce pressure on other health services, by reducing costs associated with unnecessary use of other more expensive healthcare services, such as visits to GPs and accident and emergency departments (EDs) at hospitals. In the year 2006 to 2007, it was reported in the United Kingdom (UK) that there were 57 million consultations with GPs involving a minor ailment, which had an estimated cost of £2 billion per annum [6]. In addition, a separate UK-based study found that of 353 observed GP consultations, 31 % were for minor ailments, of which 59 % could have been managed in a community pharmacy [15].

Research undertaken in Australia found that if the resources devoted to minor ailments were dealt with through community pharmacies, this redirection of resources could potentially free-up the equivalent of 500 to 1,000 full time GPs to treat more serious health problems [16]. In addition to GP visits, estimates have been made of the minor ailments managed in EDs and afterhours clinics, which could have been managed by a pharmacist [17–20], ranging from 5.3 % [17] to 8 % at EDs [19], and 28 % of adult attendances at afterhours primary care centres [20].

The potential for community pharmacists to act as providers of triage services has started to be recognised, and community pharmacy triage services are emerging in a number of countries. For example, the Swiss Pharmacists’ Association has launched netCare in a select number of pharmacies [21]. netCare is a primary triage service using a structured decision-tree for 24 common conditions, where pharmacists can request a real-time video consultation with a doctor if necessary. In addition, minor ailment schemes have been implemented, for example, the Community Pharmacy Minor Ailments Scheme (MAS) [6, 21], which began in Scotland and is now available at some pharmacies across the UK. These minor ailment schemes have elements of triage within their structure and formalise the primary health care role of the community pharmacist for certain minor ailments, whereby designated patients can consult a pharmacist and, if necessary, obtain a pharmacist-prescribed medication from a limited formulary [21]. In Canada, two provinces (Nova Scotia and Saskatchewan) added minor ailments as an expanded aspect of practice in 2011. This new legislation broadened pharmacists’ scope of practice, enabling them to prescribe certain medications for minor self-limiting and self-diagnosed ailments from a list of agents previously only able to be prescribed by a doctor [22].

The aim of this review is to explore the potential for community pharmacy provision of triage services. Specific objectives were:

- To explore whether key components of triage services can be identified in literature surrounding community pharmacy
- To explore the evidence for the feasibility of implementing community pharmacy triage services (CPTS)
- To evaluate the evidence of appropriateness of such services

## Materials and methods

### Working definition of triage

For the purposes of this paper, we used a definition of community pharmacy triage reported by Chapman et al. [23], In their report they described triage in this way “The provision of advice about how best to manage health issues – whether with a medical product or device or with non-drug measures, whether to seek assistance from a doctor or other health professional, and with what sense of urgency – is a primary health care service commonly provided by community pharmacies”.

### Definition of appropriateness

This review aimed to evaluate the evidence of appropriateness of CPTS. For the purposes of this study, appropriateness was considered in the light of clinical appropriateness and acceptability by other health professionals and patients.

### Search strategy

We performed systematic searches in MEDLINE, EMBASE and International Pharmaceutical Abstracts (IPA) databases from 1980 to March 2016. The search strategy was designed to retrieve studies conducted on triage-like services in community pharmacy settings. Triage in community pharmacy is a relatively new and developing concept that does not have a clear definition; an initial search revealed that published literature on community pharmacy seldom uses the word triage; therefore, this review used several synonyms for the relevant activities that comprise our working definition of triage in community pharmacy to capture articles related to this concept.

**Fig. 1:**
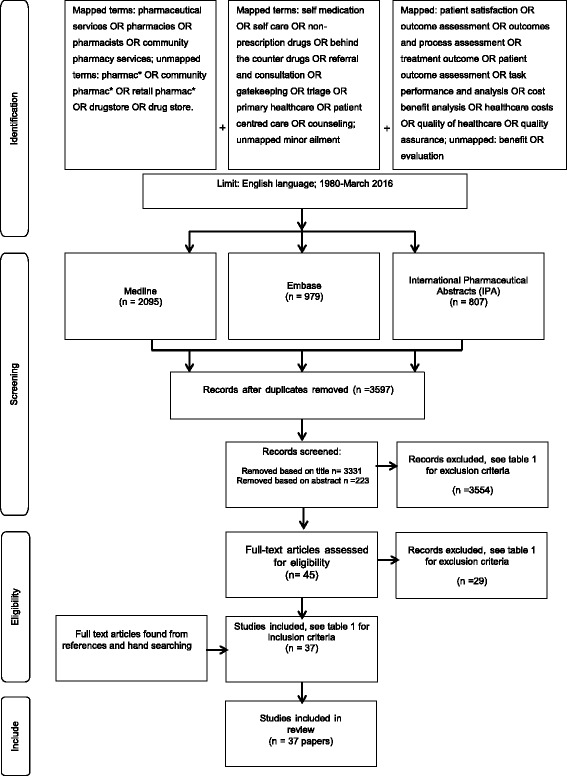
The process of identification, screening and inclusion of papers for this review

Our search included both mapped and unmapped terms, which are illustrated in Fig. 1. In addition, the following text words and MeSH/EMTREE terms were used to identify additional relevant papers: (Mapped terms: pharmaceutical services OR pharmacies OR pharmacist OR community pharmacy services; unmapped terms: pharmac* OR community pharmac* OR retail pharmac* OR drugstore OR drug store) AND (Mapped terms: self medication OR self care OR non-prescription drugs OR behind the counter drugs OR referral and consultation OR gatekeeping OR triage OR primary healthcare OR patient centred care OR counselling; unmapped minor ailment).

### Study selection

Inclusion criteria were formulated in relation to the research aims. First, papers were included only if they referred to community pharmacy settings and included a triage service (as defined above) in patients with a first presentation of a medical complaint. We excluded studies that were not written in English, did not have a full text article available, reviews, commentaries and letters to the editor. We also excluded studies that focused on services for monitoring chronic/long term conditions or were focussed on prescription services.

### Data extraction and analysis

Two researchers (LC, JM) independently extracted study characteristics, using an extraction table. One researcher (LC) compared all extracted data and discussed discrepancies with other researchers (JShe, MM) when necessary. A summary of the data extracted from the studies is presented in Table 1. This includes the study design, aims, measurements taken, types of conditions, number of referrals and a summary of results. In addition, we recorded whether each study included the characteristics of community pharmacy triage, based on our working definition, in their study description: i.e. contact with the patient or caregiver, questions asked, urgency and level of care decided, advice given and decision made to treat or refer. Evidence of appropriateness in decision making, appropriateness of referral, adherence to referral advice, and the recommendations from the authors were extracted from the studies.

**Table 1:** Overview of identified studies

Author (year) country [reference]	Key aims	Study design	Measurements	Participants	Proportion referred (%[n])	Summary of results	Characteristics of community pharmacy services that match the working definition of triage				
							Contact between community pharmacy and the patient or caregiver/proxy	Questions are asked to determine the diagnosis	Urgency and level of care decided	Advice given	Decision made to treat or refer
Alkhatib et al. (2015) Australia [44]	To evaluate pharmacists’ management of eye infections following the reclassification of ophthalmic chloramphenicol.	Cross-sectional postal survey to a randomized sample of community pharmacies.	Agreement or specific information by the pharmacist on: 1. Provision of ophthalmic chloramphenicol 2. Protocol and training 3. Pharmacist views 4 Demographics from recall	119 responses from pharmacist managers/ proprietors	Not recorded	Pharmacists’ capability to treat acute bacterial conjunctivitis was improved and pharmacists felt that there was better utilisation of their professional skills. There was improved access to treatment options for patients. More education and training was signalled by some and use of protocol differed by age group. More sold OTC in larger pharmacies in metropolitan areas, no change in number of prescriptions for chloramphenicol. No evaluation of service.	✓	✓	✓	✓	✓
Baqir et al. (2011) UK [6]	To assess what action patients using MAS would have taken if the MAS had not been in place and to approximate the net cost impact.	A cost minimization analysis of submitted claims data. One item questionnaire for consumer.	Patients were asked what action they would have taken if the MAS was not in place. The calculated net cost impact of the MAS using standard health-care reference costs.	396 patient claims were recorded	Not recorded	Savings of NHS resources over 1 month equated to £6739.01. Estimation of which resources would be used if not in place identified GPs and EDs as the next port of call.	✓	✓	✓	✓	✓
Berger et al. (2005) Germany [37]	To assess the quality of patient counselling in community pharmacy and evaluate a new method of feedback.	Observational study using pseudo-customer methodology	All aspects of the interview, recommendation and advice.	49 community pharmacies	90 % [27] of cases that warranted referral	More assessment was conducted when patient presented with symptoms than a product request. Some appropriate self-medication advice provided in 74 % of visits, usually not sufficient. One of the two cases the optimal decision was referral, whereas the other case medication and advice was sufficient. 90 % of cases that warranted referral were referred but only 30 % with necessary urgency.	✓	✓	✓	✓	✓
Bilkhu et al. (2013) UK [34]	To determine and quantify questioning and management of a patient with presumed allergic conjunctivitis.	Observational study using pseudo-customer methodology	All aspects of the interview, recommendation and advice. The type of pharmacy staff who was involved in the consult	100 community pharmacies	14 % [13]	Average questions asked 3.5 ± 2.6. Differential diagnosis questioning and management of allergic conjunctivitis by community pharmacies in this study was lacking. Referral to optometrist comprised 2 % of the 100 pharmacies. 91 % advised on treatment.	✓	✓	✓	✓	✓
Blenkinsopp et al. (1991) UK [50]	The aims of the study reported evaluation of pharmacist used referral cards	Questionnaires completed by both pharmacist and GP	1. Usefulness and acceptability of the notification card 2. The use of the card in the reporting of suspected adverse drug reactions from the community pharmacist to the GP 3. Acceptability and value of such a card	Six pharmacies, 15 general practices in two towns	Not recorded % [120]	71 % of patients who were referred to their GP by the pharmacist did so. Overall, 12 % of cards issued were for a suspected adverse drug reactions. Their was a positive perception of the cards by all parties - patients, doctors and pharmacists. Of the referrals GPs felt 88 % of cases were referred appropriately.	✓	✓	✓	✓	✓
Bojke et al. (2004) UK [7]	To investigate the effects of an intervention to provide easier access to pharmacists for patients with minor ailments.	Analysis of consultation numbers and types. Patient minor ailment type and influencing factors.	1. Effects of the intervention on the total number of consultations by GPs and on the mix of patients seen by the GP 2. Factors affecting patients’ choices between GP and pharmacist consultations for minor ailments	1521 consultations of which 575 patients took the pharmacy option to treat minor ailment	Not recorded	The total number of GP consultations was unaffected but the intervention led to the number of minor ailments consultations decreasing. The main reason behind patient choice in consulting the GP/pharmacist was the type of minor ailment. Distance did not alter patient choice.	✓	✓	✓	✓	✓
Chui et al. (2005) Singapore [53]	To identify pharmacist’s approach in providing advice and consumers’ behaviour in self-treatment and their perception of the community pharmacist’s role in advice.	Two structured questionnaires	The pharmacists and consumers were surveyed independently using two structured questionnaires.	44 pharmacists and 181 patients	15.5 % [28] said that pharmacists had referred them to a GP.	The majority of pharmacists gave advice on self-medication to at least 10 patients per day. The majority of patients (90.9 %) were at least somewhat satisfied with advice provided.	✓	✓	✓	✓	✓
Coelho et al. (2014) Portugal [24]	To determine the prevalence of self-medication and to evaluate the clinical impact of pharmaceutical counselling.	Cross- sectional observational study	All aspects of the interview were recorded including the recommendation advice and when referred	298 patients	9.1 % [27]	51.3 % presented asking for advice, 48.7 % asking for a specific product. 9.1 % referred to GP. Follow up - After 1 week of pharmaceutical intervention, 86.8 % had a positive impact, half of referred patients made GP visit, 80 % of counselled patients had improved symptoms.	✓	✓	✓	✓	✓
Chapman et al. (2010) Australia [23]	To understand the nature and impact of primary health care that is provided by community pharmacies	Cross- sectional observational study	Consultations between customersand staff in community pharmacies. Interview with each customer post consultation and a follow-up phone call.	24 community pharmacies; 280 customers (telephone contact made with 252)	4.2 % (5.6 %) [12 direct (16 conditional)], in addition 3 % (8.3 %) [4 (11 conditional)] from proxy consultations	Most elements of consultation only took place when a customer sought advice versus a product. Most did not take the advice of referral from the community pharmacy.	✓	✓	✓	✓	✓
Driesen et al. (2009) Belgium [35]	To assess management of acute diarrhoea in an 8-month-old baby using a simulated patient scenario in a community pharmacy	Observational study using pseudo-customer methodology	This outcome was assessed against the three WWHAM questions that were defined as the most essential topics to be able to evaluate the situation	101 community pharmacies	31 % [not recorded]	The majority of pharmacists asked too few questions to adequately analyse the situation. Advice was given but insufficient counselling on medicines. 31 % referral including conditional, good counselling on dehydration. Authors reported too few questions asked to adequately assess the scenario.	✓	✓	✓	✓	✓
Erni et al. (2016) Switzerland [48]	To evaluate the impact of this new service as well as the added value for the health care system.	Cross- sectional study	Ailment, procedure of the consultation, treatment, patient information and outcomes of the follow-up call on a standardized form submitted to the study centre.	Pharmacists from 162 pharmacies performed 4118 triages.	7 % [288] (17 % required second opinion of medical practitioner)	4118 triages were completed by 162 pharmacists In 17 % of the cases the option to have a backup consultation was utilised. In follow-up calls, 84 % of the patients who were seen only by pharmacists reported complete relief or symptom reduction. Significant or complete remission was seen in 84 % of the patients triaged by the pharmacist. 9 % required another medical consultation, 7 % of patients needed further pharmacy treatment.	✓	✓	✓	✓	✓
Evans et al. (2005) UK [41]	To find out whether community pharmacy was offering appropriate advice to patients seeking advice on management of a persistent ulcer on the tongue.	Observational study using pseudo-customer methodology	The interviewer then recorded the advice given and noted whether the source was a pharmacist or a pharmacy assistant	40 pharmacists and 40 pharmacy assistants	Pharmacist: 81 % [33] Pharmacy assistant: 35 % [14]	The most appropriate outcome would be referral. Most pharmacists gave the correct advice of referral. Pharmacy assistants gave inadequate advice in most cases.	✓	✓	✓	✓	✓
Hafejee et al. (2006) UK [45]	To elucidate the range of skin problems currently encountered and knowledge to deal with these	Questionnaire survey	Pharmacists’ dermatology education, patient resources available, and the nature of the skin problems for which patients consulted them and the follow-up arrangements.	20 community pharmacists and 735 dermatological presentations	84 % if symptoms did not resolve [not recorded]	There is high number of presentations for dermatological advice, and the presentations are varied. There is a need for more focused dermatology topic teaching for pharmacists both at undergraduate and postgraduate levels. 84 % of pharmacists told patients to consult their GP if symptoms did not resolve.	✓	✓	✓	✓	✓
Hassell et al. (1997) UK [11]	Patients qualitative views on pharmacy services and roles	Ethnographic-style research study	Staff and patient interviews and non-participant observations of medicine and health interactions	Ten pharmacies, over 1000 patients interviewed and 44 telephone interviews	6 % [not recorded]	Patients used pharmacy instead of GP due to: costs, convenience, and illness seen as minor, to see if pharmacist thought they should see GP. Pharmacists play a major role in keeping minor ailments out of the GPs, and act as a referral mechanism if necessary. Follow up on a sample of the patients seen to check relief of symptoms/ resolution of problem, but outcome not recorded.	✓	✓	✓	✓	✓
Hassell et al. (2001) UK [51]	To assess the extent to which patients would visit a community pharmacy instead of a GP for management.	Intervention study	Transfer rates and reductions in general practice consultations for the 12 conditions. Prescribing outcomes and re-consultation rates.	Eight community pharmacies, 1522 patients	3.6 % [21]	37.8 % of eligible patients accepted offer of transfer to community pharmacy for consult and treatment. 3.6 % referred back to GP, 5.7 % re-consultation within 14 days. Pharmacy treatment acceptable and feasible	✓	✓	✓	✓	✓
Jiwa et al. (2010) Australia [46]	To characterize factors affect pharmacists providing a referral for patients with lower bowel symptoms to consult a general practitioner	Questionnaire	Vignettes were constructed around 6clinical variables and pharmacists were asked to describe a referral pathway.	167 community pharmacists and 1503 vignettes	69 % [1040]	Cases presented to pharmacists as vignettes. Pharmacist triage was in agreement with expert panel in 70 % of cases. Diarrhoea over referred and weight loss and rectal bleeding under referred.			✓		✓
Jiwa et al. (2012) Australia [49]	To develop a tool to assist community pharmacists to triage patients presenting with cough	Assessment tool development and pilot of tool	Leicester Cough Questionnaire; Pharmacy Cough Assessment Tool including referral and follow up	Four pharmacies and ninety-nine subjects	37 % [37] (however 18 more qualified for referral)	The tool identifies patients with cough who might benefit from medical advice and may feasibly be used as an initial screening tool in the community pharmacy setting. 7/37 participants who were referred to their GP could be confirmed to have done so. Two were prescribed antibiotics; one was referred for a chest X-ray and one to a specialist.	✓	✓	✓	✓	✓
Kippist et al. (2011) Australia [39]	To investigate how community pharmacists respond to complaints of acute insomnia from people who seek self-treatment and determine the factors affecting this response.	Observational study using pseudo-customer methodology	Supply/non supply of a sleep aid and scores for pharmacists for skills in eliciting information prior to supply of medication	100 community pharmacies	4 % [4] (24 % of cases overall made some type of referral incl to revisit if no resolution)	Many pharmacists are responding appropriately. The most appropriate outcome would be non-supply of medicine A product was supplied in 96 % of visits; conventional medicines in 65 % of cases, and herbal/ homeopathic medicines 31 %.	✓	✓	✓	✓	✓
Krishnan et al. (2000) Germany [33]	To determine whether patients with dyspepsia had improved outcomes in quality of life scores comparing an intervention sand a control pharmacy	Observational and questionnaire	Quality of life scores before and after self-medication. Quantitative and qualitative evaluation of pharmacist advice	36 pharmacies 198 patients	10.8 % [21] and 68.7 % conditional referrals	Overall counselling in trained pharmacies was better than non-trained pharmacies. In general patients were asked comprehensive questions and provided with advice. Longer consults were associated with more satisfied reports. However, some pharmacists did not provide sufficient warning for those who were at risk. Drug related problems were not addressed sufficiently.	✓	✓	✓	✓	✓
Mansell et al. (2015) Canada [22]	To determine whether patients prescribed such treatment by a pharmacist symptomatically improve within a set time frame.	Online questionnaire for patients	Demographics, condition, pathway to encounter, outcome including satisfaction and further consultation needed.	Ninety pharmacies and125 participants.	Not recorded	Trust in pharmacists and convenience was the most common reasons for choosing a pharmacist. 27.2 % would have chosen a physician or ED otherwise. Satisfaction with the pharmacist and service was strong; only 5.6 % felt a physician would have been more thorough. The condition significantly/completely improved in 80.8 %; 4 % experienced side effects.	✓	✓	✓	✓	✓
Marklund et al. (2003) [32] Sweden	To assess whether pharmacists make appropriate choices with patients with dyspepsia.	Assessment of referral cards	Demographics, reason for referral, assessment of referral	132 patients	Not recorded [132]	Of all of the patients who were referred, the assessors agreed that 90 % of the patients should have been referred to their GP.	✓	✓	✓	✓	✓
Martin-Morales et al. (2013) Europe [Greece and Spain] [28]	To assess pharmacists’ ability to detect erectile dysfunction and encourage patients to seek medical evaluation.	Cross-sectional observational study in two countries	Proportion of men with a SHIM score ≤ 21 and, of those, the proportion who visited a physician and credited the pharmacist for their visit.	451 patients	77 % [348]	First health care professional approached - 50 % pharmacist, 18 % GP. Follow up phone call to verify the quality of the patient education provided and whether they visited GP. Less than 1/3 referred to GP had visited	✓	✓	✓	✓	✓
Maunder et al. (2005) UK [63]	To assess the advice given by pharmacists on oral health and the role of pharmacists in oral healthcare services	Questionnaire	Pharmacy characteristics, products available, knowledge of pharmacists and promotional activities	17 pharmacies	94.1 % of cases to see the dentist and, 23.5 % to see the GP [not recorded]	Most common presentations during data collection was for ulcers and toothache/pain. Advice was given to see a dentist/Dr. Albeit pharmacists had little knowledge of the dentists in the area or emergency arrangements. Pharmacists were interested in having protocols for management of oral health care.	✓	✓	✓	✓	✓
Mehuys et al. (2009) Belgium [30]	The role of the pharmacist in triage related to upper gastrointestinal presentations	Questionnaire-based referral tool	1. Nature of GI symptoms that people intend to self-medicate 2. Prevalence of alarm symptoms 3. Adherence to referral advice 4. Self-reported efficacy	592 patient consultations	21 % [124]	Only 51.7 % of the customers, who were referred, adhered to that advice. Overall 48.7 % of people reported symptom relief and of those given OTC treatment 95.1 % reported relief of symptoms.	✓	✓	✓	✓	✓
Parmentier et al. (2004) UK [52]	To evaluate a scheme offering pharmacy referrals for minor ailments in a refugee community.	Intervention study	The presenting minor ailment and corresponding medication as recorded by the pharmacist.	2 community pharmacies, 184 refugees	1.1 % [2]	200 vouchers were distributed to 184 refugees. Of all the referrals, there were two clients who were referred to the GP and two advised to see the GP if symptoms persisted.	✓	✓	✓	✓	✓
Phillips et al. (2001) UK [27]	Use of community pharmacy versus general practice was acceptable as the first point of call for head lice.	Before and after training study and questionnaires to health professional and patient	Before and after training where pharmacists were asked to record head lice consults	571 patient consultations	Not recorded	Patients treated for head lice by pharmacist rather than GP. Estimated savings during study period of up to £52000.	✓	✓	✓	✓	✓
Ralph et al. (2001) UK [47]	To assess the ability of pharmacists to appropriately manage a range of genital symptoms	Questionnaire on case-based scenarios	Pharmacist perceptions on their ability to manage a range of genital symptoms and their knowledge of genitourinary services	28 community pharmacies	4–100 % dependant on the condition, some with OTC products	Range of symptoms/conditions surveyed. Focus on pharmacist knowledge of genitourinary services nearby - low. Showed that many pharmacists know when to refer STDs, but more education on services to refer to needed.			✓		✓
Rutter et al. (2004) UK [36]	To determine whether an appropriate course of action was taken by UK community pharmacists for cases of headache and abdominal pain	Observational study using pseudo-customer methodology	All aspects of the interview, recommendation and advice	28 community pharmacies	53.6 % [15]	Referral was expected outcome - advised in 53.6 % of cases. Most questions asked were relevant (66 %) but inadequate histories taken.	✓	✓	✓	✓	✓
Schneider et al. (2011) Australia [38]	Evaluation of pharmacist assessment and triage when appropriate for chronic cough.	Observational study using pseudo-customer methodology	Demographic details, assessment questions, and advice provided.	155 community pharmacies	38 % [59] (36 % of these provided OTC supply also)	Referral was ideal outcome based on symptoms; only 38 % of cases were referred. Adequate assessment increased likelihood of referral. Consultations conducted by pharmacists were more likely to lead to appropriate outcome.	✓	✓	✓	✓	✓
Scully et al. (1989) UK [43]	To assess the advice offered by pharmacy staff with a potential oral carcinoma	Observational study using pseudo-customer methodology	Advice and recommendation.	57 community pharmacies	8.8 % [5]	Referral was the ideal outcome based on the symptoms; only 8.8 % of consultations were advised to see a doctor (*n* = 4) or a dentist (*n* = 1), after medication advice.	✓	✓	✓	✓	✓
Symonds et al. (2011) Europe [UK, Germany, Czech Republic and Spain] [29]	To determine if community pharmacists could appropriately recommend suitability for supply of sildenafil 50 mg for the treatment of erectile dysyfunction	Cross-sectional observational study of natural patients	Concordance rate between pharmacist and physician recommendations.	53 pharmacists, 13 physicians and 346 participants	Not recorded	Agreement between pharmacist, GP and specialist recommendations assessed. 90 % of cases specialist agreed pharmacist gave an acceptable recommendation.	✓	✓	✓	✓	✓
Varela-Centelles et al. (2012) Spain [42]	To assess whether pharmacies and herbalist’s shops were offering appropriate advice for patients seeking guidance on a potentially malignant oral lesion	Observational study using pseudo-customer methodology	Individual interaction with the interviewee according to a previously prepared script and details were recorded	306 community pharmacies and 154 herbalist shops	27.5 % [84] referrals and 36.3 % [111] referrals in addition to OTC sale	The most appropriate outcome was referral. Community pharmacies referred more than herabalists. Pharmacy assistants were more likely to recommend OTC remedies (55.6 % vs. 13 %) and significantly less likely to refer than were pharmacists.	✓	✓	✓	✓	✓
Vella et al. (2009) Malta [26]	To design two protocols to help pharmacists care for consumers seeking treatment for headache and back pain and assess pharmacists’ management of these conditions	Observational study using pseudo-customer methodology	Data for each case and divergence from protocol	10 pharmacies and 212 patient interventions	Not recorded	Compliance higher when pharmacists responded to symptoms than when product asked for by name - less advice given when product requested.	✓	✓	✓	✓	✓
Watson et al. (2015) UK [64]	To compare health-related and cost-related outcomes of consultations for symptoms suggestive of minor ailments in EDs, GPs and community pharmacies.	Cross-sectional study	1. Whether health-related and cost-related outcomes differ between settings. 2. Whether satisfaction with index consultation is associated with health-related outcomes. 3. What factors (triggers) influence patients’ choice of care setting.	377 patients participated, recruited from EDs (81), general practices (162) and community pharmacies (134).	Not recorded	Symptom resolution was similar across all three settings: ED (37.3 %), GP (35.7 %) and pharmacy (44.3 %). Mean overall costs per consultation were significantly lower for pharmacy	✓	✓	✓	✓	✓
Watson et al. (2016) UK	To assess what factors predicted whether the supply of a guideline compliance for the supply/non-supply of non-prescription medicines.	Observational study using pseudo-customer methodology	The questions asked during the consultation and the outcome of the consultation	351 patient visits (but some missing data)	Not recorded	WWHAM questioning was associated with appropriate outcome. After adjusting for WWHAM scoring the outcome was twice as likely to have an appropriate outcome than other consultations. The likelihood of an appropriate outcome increased if the consultation was conducted by the pharmacist	✓	✓	✓	✓	✓
Westerlund et al. (2003) Sweden [31]	To measure the outcomes of a counselling model for dyspepsia	Observational study using pseudo-customer methodology	All aspects of the interview, recommendation and advice and a follow up interview by research staff	33 pharmacy staff and 319 patients	12 % [39]	A counselling model to discover and resolve problems related to symptoms and drug use appeared to have a favourable impact on outcomes. Patient outcome: Only 1/5 customers referred contacted GP. 2/3 reported feeling better following self- care advice.	✓	✓	✓	✓	✓
Westerlund et al. (2007) Sweden [25]	To assess the quality of self-care from pharmacist using IT clinical guidelines	Cross-sectional study, where outcomes were reviewed by a doctor and follow-up with the patient occurred	Questions asked and information given Follow-up with the patient	10 pharmacists and 250 customers	Not recorded	Self-care counselling when supported by IT-based clinical guidelines is high. Independent assessment found a 97.6 % of the consultations were appropriate. Follow-up found that there was a favourable feedback from patients. Referrals were not included in this study	✓	✓	✓	✓	

## Results

### Screening, selection and included studies

A diagrammatic depiction of the search strategy is included in Fig. 1. The searches in MEDLINE, EMBASE and IPA resulted in a total of 3597 titles. Studies were excluded if they were not related to community pharmacy triage or did not report outcomes related to patients. Duplicates were also excluded. The remaining studies (*n* = 37) reported aspects of triage in community pharmacy between 1980 and 2016 (Table 1). The studies were undertaken in the UK (*n* = 16), Europe (*n* = 13), Australia (*n* = 6), Canada (*n* = 1) and Singapore (*n* = 1).

Three main methodologies were used across the studies. Twenty-two of the studies in this review were cross-sectional observational studies with natural patients. Ten studies used a pseudo-patient methodology, which in our review was defined as studies where a trained person presented to a pharmacy asking for advice or a specific product as part of a pre-determined case, and consultation was recorded and feedback given to the pharmacy. Lastly, questionnaires completed by healthcare providers and/or patients (*n* = 5) were also used where they described the aspects of a community pharmacy triage service.

#### Types of conditions

Thirteen studies included any minor ailment in community pharmacies across a given time period, whilst others presented results on specific conditions across a time period (*n* = 24). Observational studies of natural patients evaluated measures surrounding non-specific minor ailment presentations [11, 23–25]. Those that focused on specific condition presentations were: headache [26], back pain [26], head lice infestations [27], two studies focussing on erectile dysfunction [28, 29] and four on gastrointestinal presentations [30–33]. All studies that used the pseudo-patient methodology focussed on specific conditions: allergic conjunctivitis [34], diarrhoea in an infant [35], abdominal pain [36], a gastrointestinal presentation [37], headache [36, 37], cough [38], insomnia [39], vaginal thrush [40] and three studies looked at ulcers/lesions in the mouth [41–43]. Four of the questionnaire-based studies investigated specific conditions: chloramphenicol use for bacterial conjunctivitis [44], dermatological conditions [45], lower bowel conditions [46] and genital conditions [47].

#### Evidence for decision making

##### Appropriate diagnosis

Appropriate decision-making with regards to treatment or referral requires eliciting a patient’s relevant history via questioning. The appropriateness in decision making was evaluated by two main methods, observing community pharmacy staff actions with the use of specific guidelines or protocols and observing community pharmacy staff actions without their use.

Ten of the studies used current or newly developed guidelines which covered asking appropriate questions and differentially diagnosing presenting conditions, and identifying requirements for referral [25, 26, 28–32, 44, 48, 49]. Other studies evaluated decision-making by recording the number of questions asked and comparing them with a pre-determined list of questions [33, 34, 36–40]; and/or the use of mnemonics such as WWHAM (Who is it for? What are the symptoms? How long? Action tried? Medications taking?) [24, 35, 40].

When a guideline or protocol was used, accuracy in identifying the presenting condition was high with concordance rates ranging from 70 % to 97.6 % [25, 28, 29, 32]. In comparison, in studies where no specific guidelines/protocols were used, the authors of those studies concluded that too few questions had been asked to obtain sufficient information to undertake a valid analysis [34–36]. For example, results from the study by Berger et al. [37] found that 95 % of community pharmacy staff asked at least one question to assess the diagnosis in patients presenting with a condition, but only 47 % in a case where a specific product request was requested.

Fifteen studies evaluated the appropriateness of the decision made to treat or refer. The studies that used pseudo-patients compared the interaction with the ‘patient’ to predetermined optimal outcomes [34–39, 41–43]. Bilkhu et al. [34] found that the differential diagnosis was lacking in community pharmacy, whereby questions were not asked to distinguish the different types of conjunctivitis. In addition, some studies found that too few questions were asked to adequately assess the presented situation [34–36]. Schneider and colleagues [38] and Watson and colleagues [40] found that the likelihood of adequate assessment increased with the number of questions asked.

In six of the natural patient studies, another health professional reviewed the outcome [25, 29, 32, 46, 47, 50]. Marklund et al. [32] had a GP assess all referrals related to dyspepsia that were recorded by pharmacists; the study found that in 90 % of cases the GP agreed that the patient needed to be referred to the GP for either a prescription, or a medical examination. Westerlund and colleagues [25], had an independent doctor assess the self-care advice given by the pharmacist and found that it was appropriate in 97.6 % of cases. In the study by Blenkinsopp and colleagues a notification card was used to improve the communication between GPs and pharmacists. If the pharmacist decided that a patient should be referred to the doctor, a notification card was completed. The card was given to the patient to take with them to their doctor and a copy was stored at the pharmacy for their records. The results showed that 88 % of the referrals were appropriate according to the GP [50]. In a separate study by Symonds et al. the medical specialist agreed with 90 % of the recommendations made by the pharmacist after a follow-up assessment [29].

In the questionnaire-based studies [46, 47], cases were given to the pharmacist who then had to make a decision on the necessity to refer. These decisions were then evaluated by a medical expert. Jiwa and colleagues [46] found a 70 % agreement between an expert panel and the pharmacist and Ralph et al. [47] reported that “many pharmacists were able to manage sexual health problems adequately”.

Between 66 % and 95.1 % of patients reported symptom relief or resolution in studies using a guideline or protocol [25, 30, 31, 48]. In the study that did not use a guideline or protocol, 86.8 % reported symptom relief or resolution [24]. In the study by Krishnan et al. [33] patients who presented with dyspepsia were contacted at 7 days post consultation with the pharmacist. One group of pharmacies had a training intervention on guidelines for counselling of patients with dyspeptic disorders and another was a control group of pharmacies who did not have this training; patients who attended both control and intervention pharmacies reported an improvement in quality of life scores at day seven [33].

#### Referral rates, appropriateness of, and adherence to advice of referral

##### Referral rates

All studies, except two (*n* = 35), discussed the referral of patients to other healthcare providers by pharmacists or other community pharmacy staff. In addition, 27 studies (see Table 1) documented either the number of patients referred or the proportion of patients referred.

There was a wide variation in the proportion of patients referred to other health services after a pharmacist or community pharmacy staff consultation. When considering the referral rate in the natural patient studies which included any minor ailment presentation, a range of 6 % [11] to 9.1 % [24] was reported. When considering the condition-specific studies this range is much wider, varying from 12 % [31] for a study on patients presenting with dyspepsia to a 77 % referral rate in erectile dysfunction cases [28].

Nine studies used pseudo-patients and documented referral [34–39, 41–43]; seven of the studies used one scenario, and the other two had two different case scenarios [36, 37]. The most appropriate, predetermined outcome in eight of the cases used in these studies was referral [36–39, 41–43] and the number of recorded patient referrals ranged between 8.8 % [43] and 90 % [37]. Three studies consisted of patient scenarios that were considered to be appropriately managed by a community pharmacy staff member; in one study no referrals were recommended [37], and the remaining two reported referral rates of 14 % [34] and 31 % [35].

In most of the studies where referrals occurred, patients were referred to a GP, but there were instances discussing referral to other health professionals, dentists in particular [41–43].

##### Adherence to referral advice

Five studies included follow up with the patient, to evaluate what proportion had taken the advice of the pharmacist to visit another health professional. In four studies, [24, 28, 30, 31] 20 %–51 % of patients had taken the advice of the pharmacist. One study found 71 % patients acted on the advice of the pharmacist; in this case a referral card had been given to the patient [50].

#### Reverse referral interventions

Whilst some studies involved patients presenting at the pharmacy directly, others described a reverse intervention service. These services offered a patient, who was seeking an appointment with a GP or nurse for treatment for a minor ailment, the option of a consultation with the community pharmacist instead. In such instances the community pharmacist could refer the patient back to the GP when necessary [7, 51, 52]. Hassell and colleagues found that the referral rate back to the GP was only 3.6 % [51] in one of their studies and 6 % [11] in the other. One study investigated refugees approaching either the nurse, support worker or reception staff at the refugee hostel about a minor ailment. Instead of being given an appointment with a GP, they were offered a voucher which they could exchange at a community pharmacy for an appropriate over the counter medication free of charge, after a consultation with the pharmacist [52]. This study had a low number of referrals (1.1 %) back to the GP [52].

#### Recommendations from study authors

Twenty seven studies included in this review noted recommendations on community pharmacy, based on their findings. These are summarised below.

##### Additional pharmacy staff education or training

Increased education, training or support for community pharmacy staff was suggested in eight of the studies in [33, 34, 39, 41, 42, 44, 45, 47]. In most cases, the recommendations were specific to the medical condition being studied, for example, appropriate advice for sexual health [47] and insomnia [39], differential diagnosis of ocular conditions [34, 44] and identifying signs of potential oral cancers with appropriate referral advice [41, 42]. In addition, Hafajee et al. recognised that there are a large number of dermatological presentations in pharmacy, and suggested increased education at both undergraduate and postgraduate levels [45].

##### Use of guidelines and protocols

Eleven of the studies suggested that guidelines or protocols be developed and used by community pharmacy [11, 22, 29–31, 34–36, 42, 46, 49]. For example, Hassell et al. [11] proposed that guidelines could be developed by pharmacists in conjunction with GPs, and a two way referral system could be established. Mehuys and colleagues [30] advocated for the use of structured questionnaires during consultations, with treatment options that ensured the recommendations made were evidence-based. Westerlund et al. [31] suggested that a model designed to diagnose and treat problems related to symptoms be used in the community pharmacy setting.

More emphasis on appropriate advice to customers was recommended by three studies [26, 35, 39]. Importantly, Vella et al., found that when customers asked for a specific product they were much less likely to be given advice on the use of that product [26]. Furthermore, the provision of patient resources and educational material was suggested [28, 29, 45].

##### Documentation and integration of care

Three of the studies made recommendations surrounding documentation of customer consultations and/or increased communication with the healthcare professional to whom the patient was being referred [48, 50, 53]. One study noted that the use of a notification card given to the patient to take to the health professional to which they were referred, improved patients following through on referral advice by pharmacists. The authors also suggested that more information could be included on this card, for example any screening measurements that had been taken, for example blood pressure, and this was being trialled [50]. Erni and colleagues [48] also proposed that future services needed better integration into the health system to ensure “its efficacy, safety, cost effectiveness and acceptance by patients”.

Documentation of patient consultations would also allow for follow-up treatment. It was suggested that there was a need for follow-up of some patients to ensure that appropriate care had been given and modification of treatment was made if necessary [28, 30].

##### Privacy and confidentiality

Phillips and colleagues [27] recognised the sensitive nature of certain conditions, and that some patients did not want to have a consultation in the pharmacy due to concerns about privacy. Having pharmacies with private consultation rooms may be beneficial for avoiding embarrassment and for ensuring confidentiality.

##### Access to the pharmacist

In the studies where it was considered that the most appropriate decisions were made [38, 42], pharmacists had conducted the consultation and thus the authors suggested that access to a pharmacist for consultations are a necessity.

##### Increased public awareness of pharmacist services

Chui et al. [53] recognised that education of the public about the services that pharmacists provide is important; in addition Hafejee and colleagues [45] noted that one inexpensive method to increase patients’ knowledge of the roles pharmacists can play in managing their skin problems was by the use of leaflets.

## Discussion

This review addressed the feasibility of, and evidence for a CPTS and attempted to identify the key characteristics of such a service that are described in the literature. This review has found that elements of a CPTS currently exist in community pharmacies; however, the components of this service may need revising as we move forward. The recommendations of the various authors identified key areas which would need to be addressed to ensure that the service is safe and effective in terms of the appropriateness of differential diagnoses and decisions to treat or refer.

Pharmacists were found to make appropriate differential diagnosis decisions in a number of studies. However, several studies that did not use guidelines/protocols noted that pharmacists or their staff did not ask sufficient questions to obtain enough information to allow them to accurately assess the patient’s condition. It is important for any consultation, whether the decision is to recommend treatment or to refer, to include adequate investigation using an appropriate number of pertinent questions. When guidelines/protocols were used this increased the appropriateness of the outcome [25, 28, 29, 32]; protocols can prompt appropriate questioning [54]. However, to optimise their use this must be coupled with training and education; Alkhatib and colleagues [44] showed that despite the high compliance with protocol use in their study, 21.8 % of pharmacists felt they required additional training. Computerised decision support systems have been trialled in community pharmacy [25], and nurse-based triage [55] with some success. If this type of protocol system were to be utilised, logistics of use would have to be further tested in a community pharmacy environment. Regardless of whether the guidelines/protocols are computer-based on not, guidelines must be reviewed on a regular basis to ensure that the recommendations are evidence-based [56].

Cost analysis was conducted in two studies based in the UK, which estimated the cost savings when patients sought advice from the community pharmacy in comparison to GPs or EDs [6, 27]. Both of these studies concluded that there would be a significant cost benefit of schemes such as the MAS.

Overall, when the appropriateness of pharmacist referral decisions was evaluated by another health care expert, a high level of concordance was found. However, to our knowledge, there have been no studies that have looked at the appropriateness of treatment provided by pharmacists for patients using community pharmacy triage-like services; studies assessing the perspectives and health outcomes for patients are also scant. Whilst OTC medications can be effective in symptom control and resolution, and many minor ailments are likely to resolve without treatment, treatment with OTC medications has the potential to mask conditions or contribute towards diagnostic delay at a GP/ED. Varela et al. [42] reported that when a pseudo-patient presented with symptoms reflective of oral cancer, few patients were appropriately referred. Similarly, Scully and colleagues [43] found that fewer than 10 % of pharmacy staff recommended referral when a patient presented with a history suggestive of oral carcinoma. In both cases, if a patient was prescribed an OTC medication, this could delay presentation at the doctor for accurate diagnosis.

In order to reduce the risk of inappropriate diagnosis and inappropriate treatment, training and the use of guidelines and protocols have been advocated [25, 28, 29, 32], to ensure that a comprehensive and relevant patient history is taken, and to guide differential diagnosis. Hassell et al. [11] proposed that guidelines could be developed by pharmacists in conjunction with GPs, and Mehuys et al. [30] highlighted the need for evidence-based recommendations within such guidelines. Erni and colleagues [48] described the netCare triage service where 24 decision trees were developed. What is not yet known is whether the implementation of these guidelines would necessarily result in compliance. Alkhatib et al. [44] found that 55.5 % of pharmacists self-reported “always” using the specified protocol for the provision of ophthalmic chloramphenicol and a further 29.4 % used the protocol “usually”. Nonetheless, 6.7 % “never” used the protocol.

Varela-Centelles et al. reported that pharmacist interactions with patients led to a higher proportion of appropriate decisions being made [42] than when consultations were with pharmacy support staff. In a study by Sheridan et al., pharmacy assistants saw themselves as being the first point of contact within the pharmacy [57], and the same study also found that pharmacists perceived pharmacy assistants as “gatekeepers” to the pharmacist. For a CPTS, it is therefore important to ensure that pharmacy support staff have adequate training, and they know when to refer to the pharmacist. The use of protocols can guide this process. However, this then raises the question of whether a future CPTS should be restricted to accredited pharmacies where staff have undertaken specific training and the pharmacies meet certain criteria.

There have been contrasting perspectives from healthcare professionals with respect to the community pharmacy’s role in the triage of minor ailments. Morris and colleagues surveyed GPs’ opinions on the treatment of minor ailments by GPs and potentially pharmacists [1]. Whilst there were favourable responses toward pharmacists in this role from some, others expressed concerns about the quality of pharmacists’ advice they did not know and only 50.9 % of GPs would recommend their patients seek advice from a pharmacist [1].

Patients have also been reported to have mixed perceptions about the role of pharmacists in healthcare. A study by Gidman et al. [58] described opinions of the public toward the role of the pharmacists and pharmacy services, including their role in the management of minor ailments. Some patients viewed the role of the pharmacist as a dispenser of medicines prescribed by the doctor and raised concerns about the incomplete nature of the services provided by community pharmacies and their lack of communication with GPs. On the other hand, others viewed pharmacists’ knowledge of OTC products to be greater than that of the GP and expressed their trust in the pharmacist as being able to competently deal with minor self-limiting conditions [58]. Erni and colleagues [48] proposed that future triage services need better integration into the health system. This notion was also highlighted by Blenkinsopp et al. [50] and Marklund et al. [32] where referral cards were used between pharmacists and GPs.

Integrated computer-based healthcare services which link pharmacy and GP data, for example, are attainable. Whilst the studies in this review did not discuss whether IT integration was available, examples do exist. In New Zealand, “Testsafe” is a medical information sharing service for certain areas of the country, which gives healthcare providers access to diagnostic test results, reports and medicines information for their patients, in addition what medications have been dispensed by community pharmacists [59]. Such a system could be used for pharmacists to report on CPTS interactions.

This review did not focus on the funding of CPTS in pharmacies; however, it is evident that cost is an important factor in considering the service’s feasibility. First of all, there is the issue of whether patients will pay for such a service. If a patient payment is required, one needs to consider whether they will use the service, in situations where GP and ED visits are free of charge, as in the UK. Conversely, in New Zealand, for example, unless you are under the age of 13, there is a cost associated with visiting a GP and thus a CPTS which is free of charge may be more attractive to patients. If no patient charge is to be made, this leaves the issue of who would fund the service.

One purpose of a recognised CPTS is to reduce the burden on other health providers such as GPs and EDs. Hassell et al. [51] found that diverting those seeking treatment for minor ailments from GPs to community pharmacies resulted in a 37.8 % reduction in GP consultations for 12 self-limiting conditions, although the overall GP workload did not decrease.

New and emerging services pertaining to the provision of advice and treatment for minor ailments, for example the MAS, are being utilised in some countries [6, 22, 52]. When questioned, patients who have used services such as the MAS, reported that if these pharmacy services were not available, they would have visited a GP or emergency services [6]. In addition, reverse referral interventions appear promising in reducing the workload of the GP for minor ailment consultations as they have resulted in few referrals back to the GP [7, 11].

An ideal CPTS needs to be one that is accessible [24] and that the public is aware of [28, 53], with sufficient resources, including competent staff that are available to appropriately question, diagnose and then either resolve or refer patients to the appropriate healthcare provider when necessary. Furthermore, communication and an interprofessional collaborative relationship between pharmacists and other healthcare professionals are integral to the success of a CPTS. Whilst a previous model developed referral cards to be taken by the patient to the referred provider [50], integrated computer-based systems may also be useful [25, 31]. Furthermore, having mutual support between GPs and pharmacists could allow for the potential of a two-way referral system [11]. In the netCare model, access to a dedicated GP to request a second opinion was available to pharmacists, which was used in only 17 % of cases [48]. This back-up consultation access may be valuable. Finally, documentation of the triage interaction is an important aspect of a potential service, and would allow for follow-up consultations to be arranged and medical notes available for re-assessment, and also allow the potential for auditing of services for quality.

It is important to differentiate community pharmacy triage from ED triage. In ED, the triage of patients involves the presenting condition being assessed for urgency and a decision on how soon treatment is required [60], and hence ED triage encompasses the management of the full range of presentations from minor to life threatening [60]. However, in community pharmacy, an additional factor needs to be acknowledged – that there are many situations in which pharmacists are not able to treat, even if they are considered relatively minor and non-urgent. Thus triage in community pharmacy is not the same as triage in ED. The importance of a clear definition of CPTS is therefore essential.

Whilst the definition used in this review (from Chapman et al. [23]) describes elements of this service, the variability in current triage services suggests that this may not be sufficient to adequately define a CPTS. Community pharmacy triage may be best described as structured service which responds to contact initiated by the patient or caregiver for advice or a specific product request. This is then followed up with appropriate questioning with the decision to treat or refer to another health practitioner. Ideally, this should then be documented in the patient’s notes held in the pharmacy and available to the GP in the patient’s electronic health record, in an integrated health system. For the presentations that do not require referral to another health care provider, treatment and advice should be recommended based on evidence-based information.

We must also bear in mind that countries worldwide differ in their provision of prescription and non-prescription medicines. There are differences in regulations about where certain medications can be legally sold and by whom. For example, in the United States [33] all non-prescription medications do not have to be sold in a pharmacy setting. This is in stark contrast to many countries in Europe where all medicines have to be sold in a pharmacy [33].

Furthermore, we chose to define “appropriateness” in the light of clinical acceptability by other health professionals and patients. However, there is lack of clarity around how or whether appropriateness could also be expanded to include other parameters outside of our criteria. This review did not focus on the funding of CPTS in pharmacies; however, it is evident that cost is an important factor in considering such a service’s feasibility, which could be a focus for future reviews.

## Conclusion

Community pharmacists are seen as the most accessible health professionals [58] and are ideally placed to provide advice on both symptom presentations and OTC medication requests [61, 62]. Some have argued that their accessibility makes community pharmacy well suited to offer extended health services, providing convenient access points to those who are unable to use other services [58]. This review explored the potential for the future provision of more formally recognised triage services by evaluating the feasibility and the appropriateness of such services. From this review it is evident that the development and use of guidelines/protocols for the management of minor ailments within community pharmacies facilitates accurate assessment of a patient’s condition with respect to whether a patient needs referral to another health care professional, and the urgency of this, or whether they can be safely treated in the pharmacy setting. Structured protocols along with adequate staff training would ensure the elicitation of a comprehensive and accurate patient history resulting in appropriate recommendations for the management of the condition. Such a service would be likely to reduce the burden on other health care providers. However, while we have highlighted the feasibility of such a service, we also acknowledge that a number of questions remain unanswered.

## Abbreviations

CPTS: Community pharmacy triage services
ED: Emergency department
GP: General practitioner
IPA: International pharmaceutical abstracts
MAS: Minor ailments scheme
